# A Depth Video Sensor-Based Life-Logging Human Activity Recognition System for Elderly Care in Smart Indoor Environments

**DOI:** 10.3390/s140711735

**Published:** 2014-07-02

**Authors:** Ahmad Jalal, Shaharyar Kamal, Daijin Kim

**Affiliations:** 1 Department of Computer Science and Engineering, POSTECH, Pohang 790-784, Korea; E-Mail: ahmadjalal@postech.ac.kr; 2 Department of Electronics and Radio Engineering, Kyung Hee University, Yongin-si 446-701, Korea; E-Mail: shka@khu.ac.kr

**Keywords:** depth video sensors, human activity recognition (HAR), body joints points, Hidden Markov Models, life-logging system

## Abstract

Recent advancements in depth video sensors technologies have made human activity recognition (HAR) realizable for elderly monitoring applications. Although conventional HAR utilizes RGB video sensors, HAR could be greatly improved with depth video sensors which produce depth or distance information. In this paper, a depth-based life logging HAR system is designed to recognize the daily activities of elderly people and turn these environments into an intelligent living space. Initially, a depth imaging sensor is used to capture depth silhouettes. Based on these silhouettes, human skeletons with joint information are produced which are further used for activity recognition and generating their life logs. The life-logging system is divided into two processes. Firstly, the training system includes data collection using a depth camera, feature extraction and training for each activity via Hidden Markov Models. Secondly, after training, the recognition engine starts to recognize the learned activities and produces life logs. The system was evaluated using life logging features against principal component and independent component features and achieved satisfactory recognition rates against the conventional approaches. Experiments conducted on the smart indoor activity datasets and the MSRDailyActivity3D dataset show promising results. The proposed system is directly applicable to any elderly monitoring system, such as monitoring healthcare problems for elderly people, or examining the indoor activities of people at home, office or hospital.

## 1. Introduction

Recent advancements in depth imaging sensors technologies have resulted in effective and inexpensive depth cameras which are actively used for 3D motion capture, surveillance systems and activity recognition [1–3]. These depth cameras sensors [4–6] produce high quality depth (*i.e.*, distance) images and are getting a lot of attention due to their potential use in human computer interaction and multimedia contents analysis. In the area of depth imaging system, a depth human body silhouette is extracted, the background is discarded, and its information contents are analyzed for its use in application areas such as human tracking, monitoring, and user recognition systems [7–9]. One major application of these systems is human activity recognition (HAR) [10–12]. Depth images could be used to monitor and recognize daily activities of residents (*i.e.*, elderly, children, or disabled people) in indoor environments (*i.e.*, smart homes, smart office and smart hospitals) and turn these environments into an intelligent living space (*i.e.*, smart environments) by making residents respond to the needs of residents [13]. Also, monitoring human activities of daily living is an essential way to describing the functional and health status of human. Thus, we set the aim of this study to develop an efficient depth-based life-logging system that monitors the activities of residents 24 h/day and comfort their life at home.

Life-logging is defined as a virtual diary that contains information records (*i.e.*, logs) such as a human's daily activities performed in indoor environments detected via HAR. Usually various sensors, such as motion sensors, video sensors, or RFID, are utilized in life-logging HAR [14–16]. The life- logging HAR system provides continuous monitoring and recording of resident's daily activities which is used for future reference to improve the quality of life by assigning life habits and patterns to users. Also, this system can help the users schedule their life (e.g., exercise time, taking medicine, office time and taking meals) according to their life style [17].

In general, HAR systems mainly deal with two different sensor devices to record the data such as video sensors and wearable sensors [18–20]. In the video-based life-logging HAR system, video cameras are used to collect video data which contains activity silhouettes of residents. In [21], the authors considered video-monitoring techniques where the human activities are analyzed in a large crowd using digital cameras and process information log as date, time and crowd situations to improve safety and security at public areas. In the wearable-based life-logging HAR system, multiple sensors are attached to the human's body parts. In [22], the authors described the system based on body-worn microphones and accelerometers attached on different parts of the body to recognize daily activities. However, it was inconvenient for the subjects to have different sensors attached to them. In addition, these sensors required wire connections which made use uncomfortable for the subject when keeping the sensors on their body for a long time.

Many researchers have adopted RGB data or depth images for vision-based HAR systems where feature sets are generated from digitized visual data or video sequences. In [23], the authors proposed different parametric models to capture the nature of shape deformations of a person's silhouette as discriminating features and provide HAR results using nonparametric models employing digital cameras. In [24], Niu and Mottaleb used motion and shape features for recognizing human activities from RGB data. Optical flow vectors are used to represent motion, eigenshape vectors are used to represent the shape of each frame in the video and HMM is used for activity recognition. In [25], the authors discussed a logging HAR system that captures videos from multiple cameras which could track and analyze human activities in real environments and then store the information in a log database for future reference. In [26], the authors used local spatio-temporal features to learn the shapes of the space-time neighborhood characteristics that are most discriminative for a given activity using different datasets and recognized activities based on a support vector machine approach.

Using depth cameras, the authors of [27] developed two state-of-the-art feature representation methods, namely spatio-temporal interest points (STIPs) and motion history images (MHIs), to create a home-monitoring oriented human activity recognition database based on a linear SVM. In [20], the authors proposed a novel depth vision-based translation and scaling invariant HAR system which recognizes home human activities in 1D feature profiles through R transform via depth silhouettes and stores the data as a log. In [28], Zhang and Parker developed a 4-dimentional (4D) local spatio-temporal feature that computes and concatenates the intensity and depth gradients within a 4D hyper cuboid, which is centered at the detected feature points and used for the task of activity recognition with the LDA model as a classifier.

Due to the occasionally problematic behavior of wearable sensors during HAR, we use video cameras for the proposed life-logging HAR system which utilizes the depth silhouettes captured by the depth camera. These silhouettes are tracked properly and produce skeleton joint points of the body parts of each activity. These joint points are computed for feature extraction and processed for training/testing routines. The system then includes training routines and recognition for life logging. The training phase of our system includes extraction of body skeletons from human depth silhouettes, identification of body joint points, and computation of motion parameters features from the body joints which are used for training of the HAR engine. Finally, after training the system, it recognizes learned activities via trained Hidden Markov Models (HMMs) for HAR and stores life log information in a database.

To the best of our knowledge, there are very few works that design and implement an intelligent activity recognition toolbox using depth cameras based on life-logging interface especially for elder people. Our proposed system is mainly focused on providing solutions to certain issues for the elderly people as: first, it provides an intelligent environment for ambient assisted living, includes scene (home/office/hospital) activity monitoring and assistance for elder people during risk factors, controls the environment and generates a log of everyday life routines which can be properly examined by doctors or nurses to improve the quality of life. Second, it provides special services for the older people such as reducing the mortality rate and overcoming extensive resource utilization. For instance, mortality rate and health problems are increasing enormously due to unsatisfactory healthcare and living alone facilities for the elderly in the European countries [29]. To deal with such circumstances, it is necessary to establish a personalized healthcare service that monitors the daily routines of elderly people, which not only reduce the mortality rate factor but also allows them to move freely as compared to institutional care or nursing homecare. According to a World Health Organization survey, the population of older people is rapidly increasing all over the world and their healthcare needs are becoming more complex, which consumes more resources (*i.e.*, human and healthcare expenditures) [30]. Consequently, our system provides services (*i.e.*, a single camera system) which overcomes the extensive resource utilization issue and improves the quality of life of elderly people. Third, the problems of similar postures of different activities are recognized by our proposed method. Our work is mainly focused on the development of a complete life-logging HAR system which provides data collection, activity training, feature extraction, modeling, recognition and log generation at a single powerful framework having a user-friendly interface and thus represents a major contribution in the field of HAR based on depth sensors.

The rest of the paper is organized as follows: Section 2 presents the life-logging HAR system methodology that includes silhouette preprocessing, body skeleton modeling, and feature generation followed by activity training and recognition using HMM. Section 3 describes the experimental results of both the conventional and proposed approaches. Finally, Section 4 concludes the paper with some comments.

## 2. Proposed Life-Logging HAR Methodology

**Figure 1.**
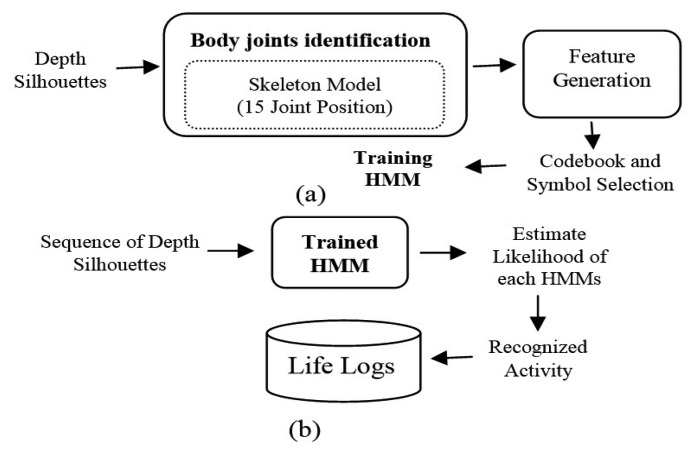
An overview of proposed life logging HAR system.

Our life-logging HAR system consists of depth silhouettes captured from the depth camera which are further processed for body joint identification, motion feature generation from the joint points and training them via HMM for HAR. An overview of the proposed life-logging HAR system is shown in Figure 1, where Figure 1a describes how to train the life logging system and Figure 1b shows how to generate life logs from the recognized activities.

### 2.1. Silhouette Preprocessing

**Figure 2.**
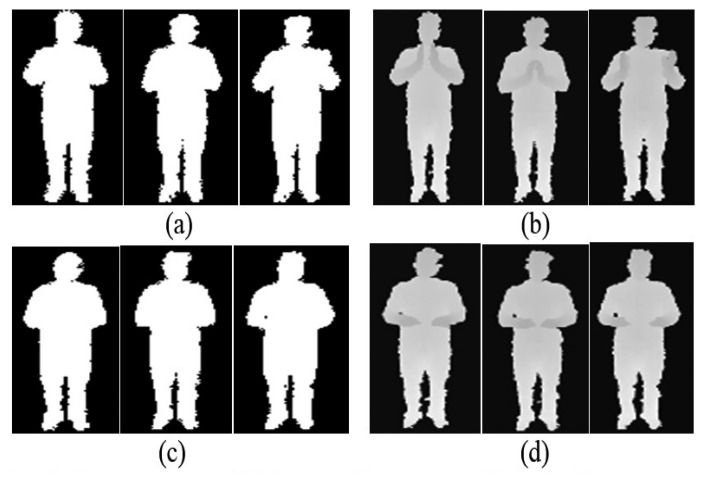
Some examples of image sequence of binary and depth silhouettes.

Initially, the system records users' daily activities from a depth camera that provides a sequence of depth silhouettes. The input sensor for our system is a Microsoft Kinect depth camera that provides RGB images and depth maps based on distance or depth information having noisy background and obstacles in the scenes. Thus, we applied background subtraction routine, tracking human depth silhouettes and produce body skeleton models for feature generation. In addition, we made a comparative study between binary and depth silhouettes to find the best silhouette nature [31–33]. After getting the depth silhouettes, we converted it into binary silhouettes using a simple threshold values. Figure 2 illustrates examples of depth and binary silhouettes, where Figure 2a,b represents the sequential binary and depth silhouettes of a hand clapping activity and Figure 2c,d represents the sequential binary and depth silhouettes of a cooking activity, respectively.

From Figure 2, it is obvious that the binary silhouettes do not seem good enough to differentiate these two different activities because the binary silhouettes deal with limited information (*i.e.*, 0 and 1 values). However, depth silhouettes clearly differentiate body parts of different activities by means of different intensity values. Depth silhouettes also deal with hidden feature information which significantly improves the recognition results especially in case of closer activities. However, during silhouette-based HAR, depth silhouettes reflect a superior recognition rate than the binary one (see Section 3.5 below). Thus, due to the better discrimination of different activities of depth silhouettes, we considered the depth silhouettes for further processing in our life-logging HAR system.

### 2.2. Training Phase of the Life Logging HAR System

For the training phase of the life-logging system, a sequence of depth silhouettes captured by a depth camera is processed to identify body joint points, the body joint points are used for generating features and the generated features are trained via HMM for the life-logging HAR system.

#### 2.2.1. Body Skeleton Model

**Figure 3.**
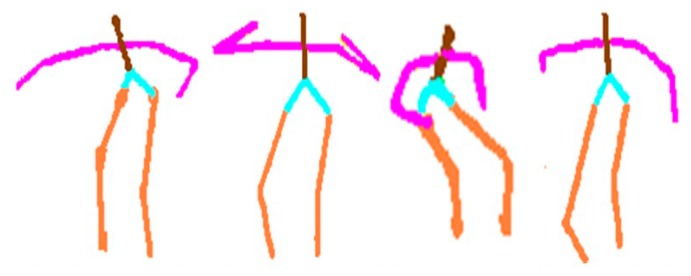
Body skeleton derived from different human activities.

From a sequence of depth silhouettes, the corresponding skeleton body models are produced. Each skeleton model is quite flexible along with any body movement activity [34,35]. Figure 3 illustrates some skeleton models of different activities in indoor environments.

#### 2.2.2. Joint Points Identification

**Figure 4.**
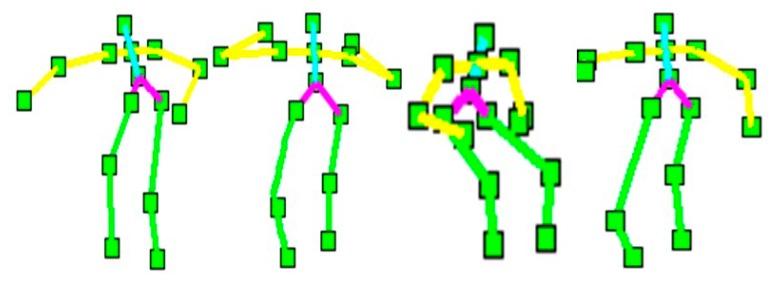
The body joint points of different human activities.

Each human silhouette is described by a rigid skeleton containing fifteen joint points. We extract this skeleton using a human tracking system provided by Open NI/NITE (PrimeSence Ltd., Washington, DC, USA) [36,37]. These fifteen joint points represent the features of the head, arms, torso, hip and legs. Each joint point has three coordinates at the frame *t*. These coordinates are normalized so that the motion is invariant to the body position, body orientation and the body size, especially during the testing interface. The depth values of joint points location in the human silhouettes [38–40] encode the presence of the features. However, to find the frontal face during human tracking, a face detector can be used to detect faces [41,42]. Figure 4 shows the body joint points of different activities of their respective skeleton models.

#### 2.2.3. Motion Parameters for Feature Representation

These features compute the position information of body joint points of motion activity. The motion parameter's magnitude of the *i*th joint point between the *t* − 1 and *t* frame can be expressed as:

(1) $${\text{M}}_{i,t}=\sum_{i=1}^{15}\sqrt{{\left(x_{i,t-1}-x_{i,t}\right)}^{2}+{\left(y_{i,t-1}-y_{i,t}\right)}^{2}+{\left(z_{i,t-1}-z_{i,t}\right)}^{2}}$$

where *x*_(*i,t*)_, *y*_(*i,t*)_ and *z*_(*i,t*)_ are *x*, *y* position and depth value respectively, at the *i*th joint point in the *t* frame. Therefore, the motion parameter's magnitudes [43] of 15 joint points becomes a vector of 1 × 15.

**Figure 5.**
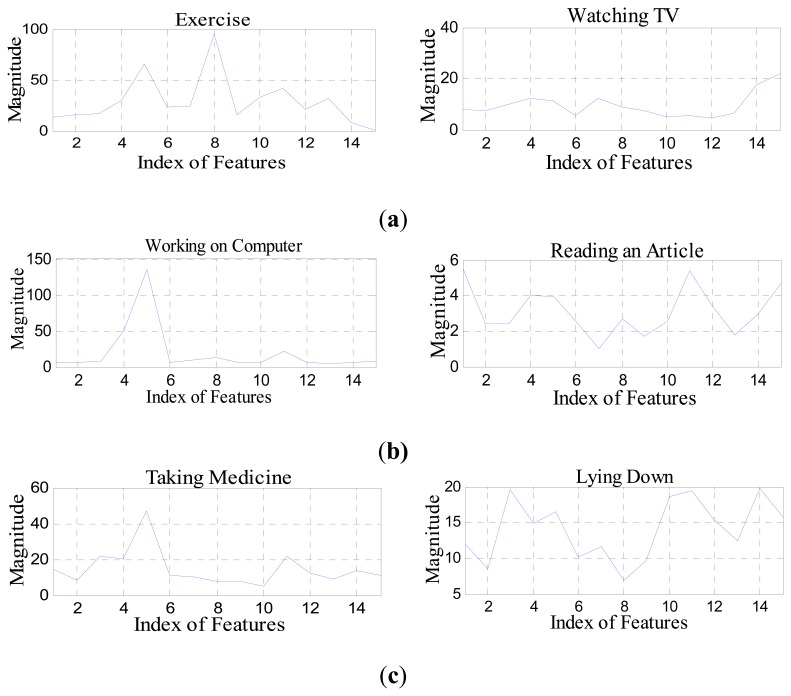
Magnitude features from the joint points of the body parts of human activities under the indoor environments. (**a**) smart home activities, (**b**) smart office activities and (**c**) smart hospital activities.

Figure 5 shows a set of magnitude features of human activities in various smart environment scenarios used in the life-logging HAR system, where (a) is exercise and watching the TV in a smart home environment; (b) is working on computer and reading an article in a smart office environment; and (c) is taking medicine and lying down is a smart hospital environment, respectively.

Directional angle features provide an estimate of angles between the motion directions for the features that are extracted from the joint points. The motion parameter's directional angles of the *i*th joint point between the *t* − 1 and *t* frame [44,45] can be expressed as:

(2) $$\theta_{i,t}^{x}=\tan^{-1}(\frac{y_{i,t-1}-y_{i,t}}{z_{i,t-1}-z_{i,t}})$$

(3) $$\theta_{i,t}^{y}=\tan^{-1}(\frac{z_{i,t-1}-z_{i,t}}{x_{i,t-1}-x_{i,t}})$$

(4) $$\theta_{i,t}^{z}=\tan^{-1}(\frac{y_{i,t-1}-y_{i,t}}{x_{i,t-1}-x_{i,t}})$$

**Figure 6.**
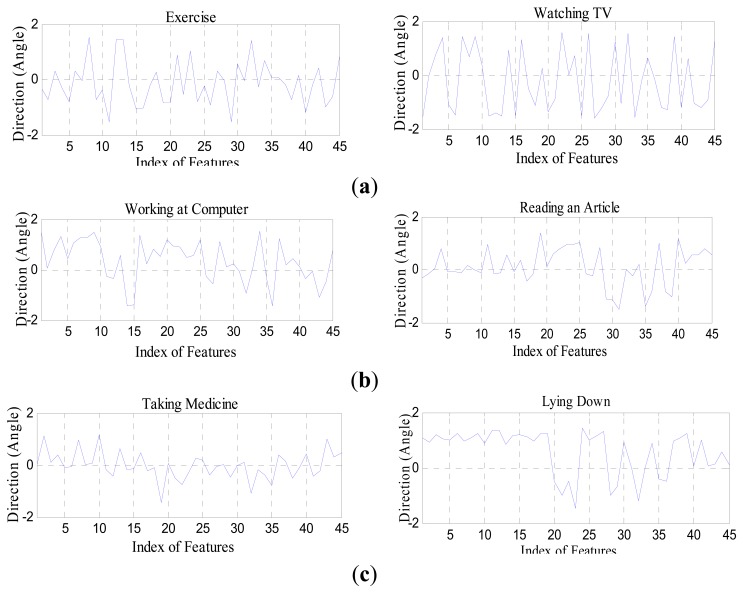
Directional angular features from the joint points of the body parts of human activities under the indoor environments. (**a**) smart home activities, (**b**) smart office activities and (**c**) smart hospital activities.

$\theta_{i,t}^{x}$, $\theta_{i,t}^{y}$ and $\theta_{i,t}^{z}$ are directional angles along the *x*, *y*, and *z* axis, respectively, at the *i*th joint point in the t frame. Therefore, the motion parameter's angles of 15 joint points becomes a vector of 1 × 45. Figure 6 shows a set of directional angle features of human activities in various smart environments used in the life-logging HAR system, where (a) is exercise and watching TV in a smart home environment; (b) is working on a computer and reading an article in a smart office environment; and (c) is taking medicine and lying down in a smart hospital environment, respectively.

#### 2.2.4. Code Representation of Motion Parameters

**Figure 7.**
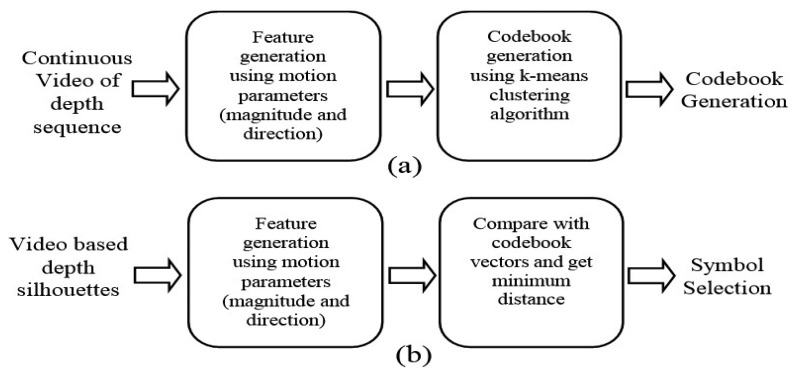
Basic steps of (**a**) codebook generation and (**b**) code selection for HMM.

These joint points features are represented by a motion parameter vector whose size is 1 × 60. Then, they are symbolized by the codebook that is generated by a k-mean clustering algorithm [46]. One input joint point feature is represented by the code that corresponds to have the minimum distance between the input joint point feature and the code's joint point feature. However, trained data get generated per each sequence and maintained by buffer strategy [47–49]. Figure 7 shows the basic steps of codebook generation and code selection of joint points features.

#### 2.2.5. HMM Training for Each Human Activity

**Figure 8.**
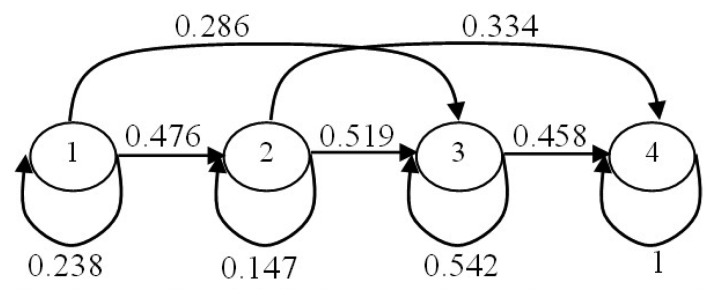
Structure and probabilistic parameters of an exercise HMM.

After obtaining the code values of motion parameters features, we train the HMM [50,51] that is used in the life-logging HAR system. In the HAR system, four-state left-to-right HMMs are used to model the human activities. While, the transition matrix is uniformly initialized according to the transition between the states. Thus, HMMs are trained based on the Baum-Welch parameter estimation algorithm [52]. Each activity is represented by a specific HMM. Figure 8 illustrates the structure and transition probabilistic parameters that are trained for the HMM of an exercise activity.

To recognize an activity, the feature vector as symbol sequence obtained from the codebook generation and symbol selection are applied on all the trained HMMs to calculate the likelihood and one is chosen having highest probability. Thus, to test a feature vector sequence *O*, the HMMs act as:

(5) $$\text{decision}=\underset{i=1,2,\ldots ,N}{\arg\;\max}\left\{P_{r}\left(O|H_{i}\right)\right\}$$

where decision is based on likelihood of *O* on corresponding trained activity HMM *H_i_*.

#### 2.2.6. Recognition and Life-Log Generation

After HMM training, the trained life-logging HAR system accepts a sequence of depth silhouettes and generates the life logs by recognizing the learned activities, which stores information such as activity type, date, time, location and number of occurrence for future references.

## 3. Experimental Results and Discussion

In this section, we explain the experimental setting. Then, we define the interface of the life-logging HAR system with both training and recognition modules. Finally, we compare the activity recognition results between the proposed and the conventional methods.

### 3.1. Experimental Settings

For the experimental setting, the smart indoor activity datasets are distributed into smart environments and 6–7 h of everyday depth video data were gathered over a week. The proposed system is evaluated in a smart room, used for three different simulated smart environments that include a home, office, and hospital where all facilities are provided according to their environment and different activities are performed with respect to their particular situation. The experiments have been carried out on a group of six elderly subjects within an age range of 58–72 years for several hours during training/testing to measure the accuracy of the detected activities recognized by the proposed life-logging HAR system. These elderly people were instructed to perform all activities freely and randomly as they would do in daily life without any instructions on how the life-logging system would interpret their movements which made it as a real living space. During the depth video recording, a fixed depth camera was hung at the top corner of the wall for each smart indoor environment separately. These depth videos are recorded at different time of different days under changing lighting conditions, from early morning, midday to late night. During feature approaches comparison, the feature vector size remained constant at 1 × 60. For training the system, a total of 40 video clips from each activity were used to build the training data. Each clip contains 20 frames. Thus, the whole training dataset contained a total of 4800 activity depth silhouettes for each smart environment, separately. In testing, six different activities of all three indoor environments are performed, giving a total of 65 video clips recorded in their respective smart environments for several hours. However, all datasets were collected in a regular indoor setting with no body occlusion from the view of the camera. To reduce unreliable depth map factors, each subject was requested to move and perform activities within a specific range of 1.3 m to 3.5 m which helped extract a reasonable skeleton size and reliable depth silhouettes. Thus, to restrict our camera path range, our datasets continuously provided ideal silhouettes of people. The proposed life-logging approach integrates efficiently in smart environments without significantly increasing computations and operated at a frame rate of 12 frames per second during testing.

### 3.2. Smart Indoor Activity Datasets

**Figure 9.**
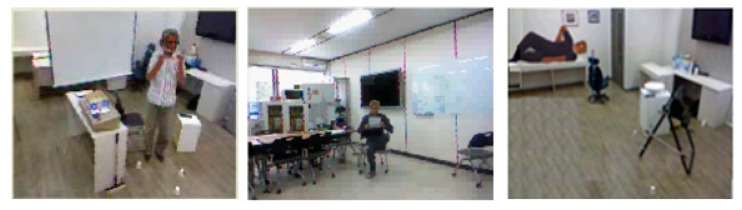
Sample from our datasets showing all three smart indoor activities environments. Smart home (**left**), Smart Office (**middle**), and Smart Hospital (**right**).

To evaluate our method, we constructed smart indoor activity datasets using the Kinect device. The videos were collected in home, office, and hospital environments, as shown in Figure 9.

**Figure 10.**
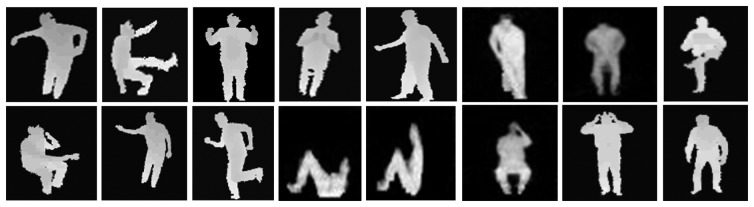
Sample frames from our proposed smart indoor activity datasets using three different environments (home, office, and hospital). Row-wise, from left: cooking, watching TV, exercise, hand clapping, walking, cleaning, sitting down, reading an article, phone conversation, presentation, rushing, lying down, getting up, taking medicine, headache, and vomiting.

**Figure 11.**
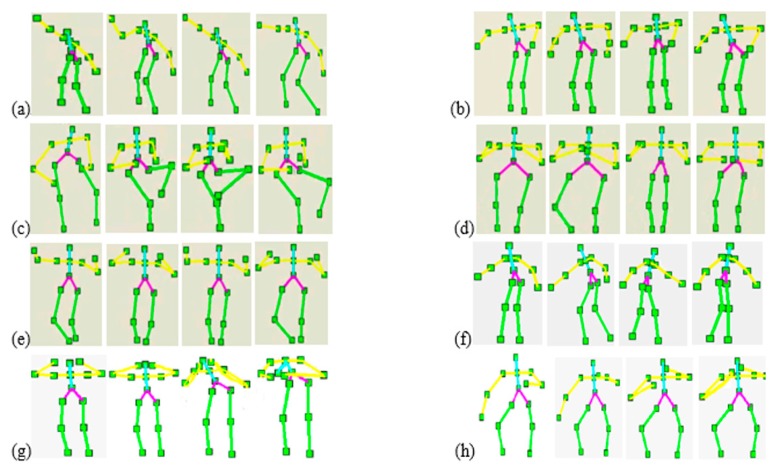
Skeleton representations that illustrate some of our results: (**a**) presentation; (**b**) cooking; (**c**) reading an article; (**d**) hand clapping; (**e**) exercise; (**f**) walking; (**g**) headache and (**h**) taking medicine.

The datasets are quite challenging because many of the activities in the datasets are highly similar to each other. Also, subjects are freely and randomly performing the various activities, thus, the trajectories of their movement make our datasets more challenging. To explain a clear picture of our datasets, Figure 10 shows some sample depth silhouettes of all three smart indoor activity environments. Furthermore, some sequential activities having skeleton representations that illustrate our datasets are shown in Figure 11.

### 3.3. Interface of the Life-Logging HAR System

#### 3.3.1. Training Phase

**Figure 12.**
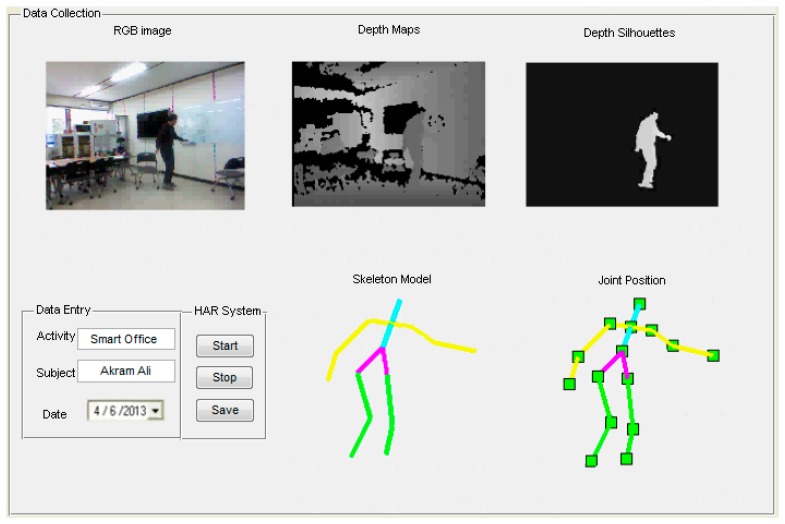
Data collection during training phase of life logging HAR system.

The training phase includes the data collection from the depth camera, computing motion parameter features and then training on the different activities via HMM. In data collection, the interface contains RGB images, depth maps having background, depth silhouettes and skeleton models with their respective joint points information, as shown in Figure 12.

**Figure 13.**
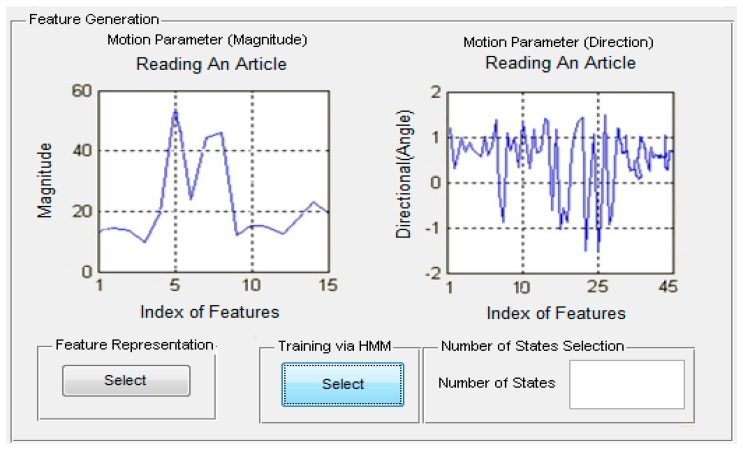
Feature generation and trained via HMM interface using different activities of life logging HAR system.

Then, the joint points information are used to compute motion parameters features based on different activities which are further trained via HMM as shown in Figure 13.

#### 3.3.2. Recognition and Life-Logging

**Figure 14.**
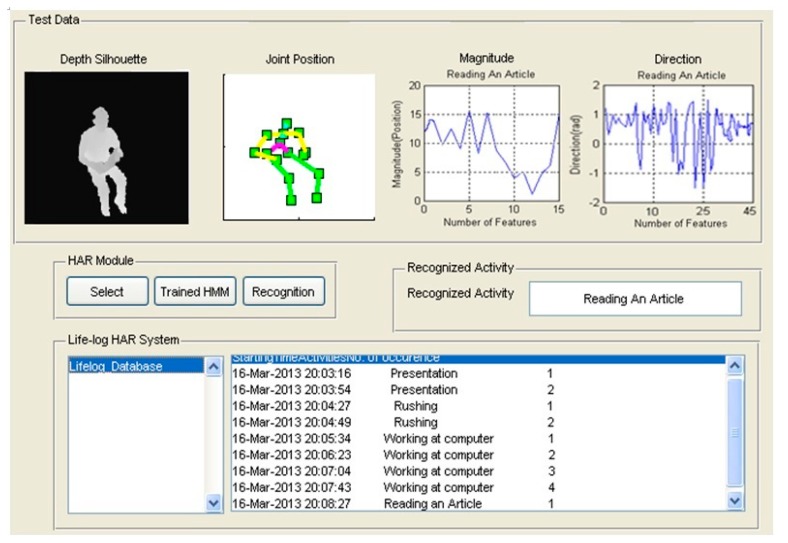
Feature generation and trained via HMM interface using different activities of life logging HAR system.

Finally, the random input activities of indoor environments are recognized using HMM and these recognized activities are stored as life logs as shown in Figure 14. Each life log includes activity name, time, date and number of occurrence in a life log database. As soon as a new activity is recognized, the life log database gets updated.

### 3.4. Comparison Study for Different Number of States of HMM

During training/testing our datasets, we selected the hidden number of states from 3 to 6 for training the HMMs and recognizing activities. Table 1 shows that we can get the best recognition rate when the number is 4. However, when we kept improving the HMM state number, there was little effect on our recognition, so we adapted four hidden states for our proposed HAR system for recognizing all indoor activity environments.

Considering the processing time of HAR for the smart home, smart office and smart hospital datasets, they were three hidden states required 13, 12, 12 fps, four hidden states needed 12, 12, 11 fps, five hidden states took 9, 8, 8 fps, and six hidden states needed 7, 6, 5 fps on a standard PC, it is concluded from above observations that HMM having a number of four hidden states still guarantees good performance both in terms of recognition rate and computational time.

### 3.5. Silhouette-Based HAR Comparison Using Proposed Motion Features

To evaluate the recognition rate of both binary and depth silhouettes, silhouette-based HAR are performed based on proposed motion features as shown in Table 2.

The recognition results of silhouettes-based HAR experiments reflect the superiority of the depth silhouettes over the binary ones.

### 3.6. Feature-Based HAR Comparison Using Smart Indoor Activity Datasets

In this experiment, we compare the proposed life logging approach using motion feature a with the approach using conventional features such as the principle component (PC) [53] and independent component (IC) features [54], where PC features of the depth silhouettes were extracted from each activity for global feature extraction and IC features were extracted from each activity for local feature extraction. The proposed system analyzed continuous activities performed in indoor environments (*i.e.*, smart home, smart office and smart hospital) by different subjects at certain hours in a day. Tables 3–5 compare the recognition results of the proposed life logging approach with the conventional methods using the depth silhouettes for smart home activities.

From Tables 3–5, it is concluded that: (1) the proposed motion features shows the highest mean recognition rate among the three different features and (2) pairs of activities such as exercise and cooking, and hand clapping and cleaning have low recognition rates in the conventional approaches due to their closer postures in smart home environments.

Tables 6–8 compare the recognition results of the proposed life logging approach with the conventional approaches using depth silhouettes in a smart office environment.

However, pairs of activities such as sitting down and a phone conversation, and reading an article and working on a computer have low recognition rates in the conventional approaches due to the complex and similar sequences in a smart office environment.

From Tables 9–11, it is concluded that: (1) the proposed motion features shows a significant improvement with recognition results as high as 90.33%, while the conventional features achieved low recognition rates of 72.25% and 78.33% in a smart hospital environment and (2) the recognition rate in the hospital environment is the lowest in all indoor environments due to its complexity and the closer postures such as headache, chest pain and vomiting.

Also, it is concluded that the proposed motion features of depth silhouettes provide better feature generation in the case of closer activities instead of global information extracted from PC features and local information extracted from the IC features.

Thus, the overall comparison among the conventional and proposed approaches showed that proposed skeleton joints features provided stronger features and a higher recognition rate than the PCA and IR transform features, respectively. In addition, the proposed HAR system achieves the best performance under various assumptions such as: firstly, the sensor (a camera) can be used from any angle for both front and side-view, however, it remains stationary during the whole activity duration. Secondly, human depth silhouettes are clearly distinguished from the noisy background. Thirdly, coordinates of the 15 joint positions are properly identified before training/testing the engine. Fourthly, an individual only performs one activity at a time rather than overlapping or concurrent ones. Lastly, feature vector-produced hidden states are meaningful sequences and mostly unique for each activity.

### 3.7. Comparison and Analysis of the Proposed HAR System with the Existing HAR Systems

The performance of the proposed HAR system is compared against six conventional methods [55–60], for all three smart indoor activity environments for HAR. All these methods are implemented by us using the instructions provided by their respective papers. Table 12 shows the depth silhouettes-based experimental results where the proposed HAR system shows a superior recognition rate and outperformed the existing HAR systems.

We compare the recognition rate of the proposed HAR system to that of [55], as [55] achieved the highest recognition rate in the above experiments among the six conventional methods where mostly methods used HMM as a recognition engine. This method [55] used spatiotemporal features which are quite sensitive to scaling, translation, and alignment of silhouettes. Thus, due to these factors, our proposed HAR system provides more satisfactory recognition rate over this conventional method [55].

In addition, the computational time of HAR taken by [55] for smart home, smart office, and smart hospital datasets is 8, 9, 6 fps, respectively. On the other hand, the proposed HAR system took 12 fps for the smart home dataset, 12 fps for the smart office dataset, and 11 fps for the smart hospital dataset. Thus, it is clearly justified that the proposed HAR system shows significant improvement over conventional methods in terms of recognition rate and computational time which make it more effective for real-world applications.

### 3.8. MSRDailyActivity3D Dataset

The MSRDailyActivity3D dataset [61] consists of daily activities captured by Microsoft Research using a Kinect device. There are sixteen activities which include drink, eat, read book, call on cellphone, write on a paper, use laptop, use vacuum cleaner, cheer up, sit still, toss paper, play game, lie down on sofa, walk, play guitar, stand up, and sit down. The total number of activity samples, in which ten subjects are involved, is 320. This dataset has been designed to cover human daily activities in a living room. Also, this dataset is quite challenging because most of the activities involves human-object interactions. During experiment results, we used a cross-subject training/testing setup in which we take out each subject (*i.e.*, leave-one-subject-out scheme) from the training set and repeat an experiment for each of them. Table 13 shows the confusion matrix of the proposed method using MSRDailyActivity3D dataset. The proposed method is also compared against other state-of-the-art methods as reported in Table 14.

In addition, other state-of-the-art methods such as the Actionlet ensemble method [61] that deals with local occupancy information based on the local 3D point cloud around each joint, Fourier temporal pyramid features, an actionlet model based on kinematic joints, and a multiple kernel learning approach provides 85.7% recognition rate and the Althloothi *et al.* method [63] which deals with multi-fused features (*i.e.*, motion of 3D joint positions and shape information) along with multiple kernel functions achieves a 93.1% recognition rate. However, the Actionlet method [61] used high-ordering features and complicated learning procedures, while, the multi-fused features [63] method used large-dimensionality features which may be unreliable for postures with self-occlusion and needs high computational times that make it impractical for long-term HAR and real-time applications. Meanwhile, our proposed HAR system is quite simple and fast for computation purposes and provides sufficient and compact feature information.

## 4. Conclusions

In this paper, we have presented an efficient depth video-based life-logging HAR system utilizing skeleton joints features generated by depth video sensors. It includes software routines for training data collection, feature generation, and training HMMs. However, our proposed HAR system utilizes magnitude and directional angular features from the joint points of the skeleton model. Once the life- logging HAR system is trained, the system can be activated to produce life logs by recognizing the learned human activities. Our experimental results showed the promising performance of the proposed life-logging HAR technique, achieving mean recognition rates of 92.33%, 93.58% and 90.33% over the conventional methods having PC features as 78.42%, 77.83% and 72.25% and IC features as 82.58%, 82.25% and 78.33% in smart indoor environments. The proposed system should be useful in many consumer application systems to monitor and generate life logs of human activities or behaviors which improve the quality of life. In future work, we aim to exploit the effectiveness of the proposed system, especially in the case of occluded regions and more complex activities by introducing some hybrid approach where the proposed system is combined with some body parts labeling method or discriminative/generative modeling [64,65] for poses to extract missing skeleton joints during occlusion which should make our human activity recognition algorithm more powerful in the future.

## Figures and Tables

**Table 1.** Comparison of recognition results using hidden numbers of states of HMMs ranging from 3 to 6 for all smart indoor activity environments, under the same setting as described in Section 3.1.

**Smart Indoor Environments**	**Different Number of States of HMMs**			

	**3-State HMM**	**4-State HMM**	**5-State HMM**	**6-State HMM**
Smart Home Activities	87.72	92.33	91.96	91.72
Smart Office Activities	88.16	93.58	93.02	92.58
Smart Hospital Activities	85.45	90.33	89.85	89.24

**Table 2.** Recognition results based on the proposed motion features using both binary and depth silhouettes.

**Indoor Smart Environments**	**Activities**	**Recognition Rate of Binary Silhouettes (%)**	**Mean**	**Recognition Rate of Depth Silhouettes (%)**	**Mean**
Smart Home Activities	Cooking	74.0	**75.67**	89.50	**92.33**
	Watching TV	80.50		97.0	
	Exercise	68.50		93.50	
	Hand Clapping	76.0		87.50	
	Walking	82.50		95.0	
	Cleaning	72.50		91.50	

Smart Office Activities	Sit Down	63.50	**72.75**	89.50	**93.58**
	Phone Conversation	71.50		94.0	
	Presentation	76.50		97.0	
	Rushing	81.0		98.50	
	Reading an Article	67.50		89.0	
	Working on Computer	76.50		93.50	

Smart Hospital Activities	Lying Down	77.0	**66.25**	96.50	**90.33**
	Get Up	71.50		88.0	
	Taking Medicine	62.50		93.50	
	Headache	58.0		85.0	
	Chest Pain	61.0		91.50	
	Vomiting	67.50		87.0	

**Table 3.** Confusion matrix based on recognition results of smart home activities using PC features of depth silhouettes.

**Smart Home Activities**	**Cooking**	**Watching TV**	**Exercise**	**Hand Clapping**	**Walking**	**Cleaning**
Cooking	**76.50**	3.50	14.50	3.50	0	2.0
Watching TV	0	**88.0**	1.50	3.0	1.0	6.50
Exercise	12.50	2.50	**79.50**	2.50	1.50	1.50
Hand Clapping	2.0	4.50	2.50	**67.50**	5.0	18.50
Walking	4.50	3.0	0	2.50	**85.50**	4.50
Cleaning	1.50	5.50	2.50	9.50	7.50	**73.50**

Mean Recognition Rate (%) = **78.42**						

**Table 4.** Confusion matrix based on recognition results of smart home activities using IC features of depth silhouettes.

**Smart Home Activities**	**Cooking**	**Watching TV**	**Exercise**	**Hand Clapping**	**Walking**	**Cleaning**
Cooking	**82.0**	0	11.50	1.0	2.50	3.0
Watching TV	0	**91.50**	1.0	2.50	0	5.0
Exercise	9.50	3.0	**81.50**	4.50	0	1.50
Hand Clapping	0	1.0	3.50	**74.0**	2.0	19.50
Walking	3.50	1.50	3.50	1.50	**87.0**	3.0
Cleaning	1.0	4.50	2.0	7.50	5.50	**79.50**

Mean Recognition Rate (%) = **82.58**						

**Table 5.** Confusion matrix based on recognition results of smart home activities using the proposed motion features of depth silhouettes.

**Smart Home Activities**	**Cooking**	**Watching TV**	**Exercise**	**Hand Clapping**	**Walking**	**Cleaning**
Cooking	**89.50**	0	7.50	0	1.0	2.0
Watching TV	0	**97.0**	0	2.0	0	1.0
Exercise	3.50	1.50	**93.50**	1.50	0	0
Hand Clapping	1.50	0	2.50	**87.50**	1.50	7.0
Walking	1.50	0	2.0	0	**95.0**	1.50
Cleaning	0	3.0	0	4.0	1.50	**91.50**

Mean Recognition Rate (%) = **92.33**						

**Table 6.** Confusion matrix based on recognition results of smart office activities using PC features of depth silhouettes.

**Smart Office Activities**	**Sit Down**	**Phone Conversation**	**Presentation**	**Rushing**	**Reading an Article**	**Working on Computer**
Sit Down	**69.0**	18.50	2.50	1.0	5.50	3.50
Phone Conversation	13.50	**76.50**	4.50	1.0	3.0	1.50
Presentation	2.50	4.0	**82.50**	5.50	3.50	2.0
Rushing	2.0	3.50	6.0	**85.0**	0	3.50
Reading an Article	5.50	7.0	2.50	1.0	**72.50**	11.50
Working on Computer	2.0	3.0	2.0	3.50	8.0	**81.50**

Mean Recognition Rate (%) = **77.83**						

**Table 7.** Confusion matrix based on recognition results of smart office activities using IC features of depth silhouettes.

**Smart Office Activities**	**Sit Down**	**Phone Conversation**	**Presentation**	**Rushing**	**Reading an Article**	**Working on Computer**
Sit Down	**75.50**	16.0	2.0	0	4.0	2.50
Phone Conversation	11.50	**81.0**	4.0	1.0	1.50	1.0
Presentation	1.0	2.50	**87.50**	3.50	2.50	3.0
Rushing	2.0	3.0	4.50	**89.50**	0	1.0
Reading an Article	5.0	7.50	1.0	2.0	**76.0**	8.50
Working on Computer	1.50	2.50	2.50	3.0	6.50	**84.0**

Mean Recognition Rate (%) = **82.25**						

**Table 8.** Confusion matrix based on recognition results of smart office activities using the proposed motion features of depth silhouettes.

**Smart Office Activities**	**Sit Down**	**Phone Conversation**	**Presentation**	**Rushing**	**Reading an Article**	**Working on Computer**
Sit Down	**89.50**	7.50	1.0	0	0	2.0
Phone Conversation	3.50	**94.0**	1.50	1.0	0	0
Presentation	1.0	0	**97.0**	2.0	0	0
Rushing	0	0	1.50	**98.50**	0	0
Reading an Article	3.50	1.0	2.0	0	**89.0**	4.50
Working on Computer	1.0	2.0	0	0	3.50	**93.50**

Mean Recognition Rate (%) = **93.58**						

**Table 9.** Confusion matrix based on recognition results of smart hospital activities using PC features of depth silhouettes.

**Smart Hospital Activities**	**Lying Down**	**Get Up**	**Taking Medicine**	**Headache**	**Chest Pain**	**Vomiting**
Lying Down	**82.50**	9.50	2.0	4.50	1.50	0
Get Up	13.50	**77.50**	3.0	2.0	2.50	1.50
Taking Medicine	5.50	3.50	**69.0**	7.50	5.0	9.50
Headache	4.0	3.50	9.0	**63.50**	6.50	13.50
Chest Pain	2.50	3.0	6.50	12.50	**67.0**	8.50
Vomiting	3.50	6.50	2.50	7.50	6.0	**74.0**

Mean Recognition Rate (%) = **72.25**						

**Table 10.** Confusion matrix based on recognition results of smart hospital activities using IC features of depth silhouettes.

**Smart Hospital Activities**	**Lying Down**	**Get Up**	**Taking Medicine**	**Headache**	**Chest Pain**	**Vomiting**
Lying Down	**84.0**	5.50	3.50	4.50	1.0	1.50
Get Up	9.50	**79.50**	5.0	1.50	3.50	1.0
Taking Medicine	3.0	1.50	**81.0**	5.0	3.0	6.50
Headache	2.50	2.0	8.50	**71.50**	4.50	11.0
Chest Pain	3.50	2.50	5.0	9.50	**76.0**	3.50
Vomiting	3.0	4.50	1.50	9.0	4.50	**77.50**

Mean Recognition Rate (%) = **78.33**						

**Table 11.** Confusion matrix based on recognition results of smart hospital activities using the proposed motion features of depth silhouettes.

**Smart Hospital Activities**	**Lying Down**	**Get Up**	**Taking Medicine**	**Headache**	**Chest Pain**	**Vomiting**
Lying Down	**96.50**	2.50	0	0	1.0	0
Get Up	6.50	**88.0**	2.0	0	2.50	1.0
Taking Medicine	0	0	**93.50**	1.0	3.50	2.0
Headache	0	1.0	2.50	**85.0**	5.0	6.50
Chest Pain	1.0	1.50	1.0	2.50	**91.50**	2.50
Vomiting	1.50	2.0	0	4.0	5.50	**87.0**

Mean Recognition Rate (%) = **90.33**						

**Table 12.** Comparison results of the proposed HAR system with some of the existing works for all three smart indoor activities environments using six daily activities each, under the same setting conditions described in Section 3.1.

**Indoor Activities Environments**	**Existing Works**						

	**[55]**	**[56]**	**[57]**	**[58]**	**[59]**	**[60]**	**Proposed HAR Method**
Smart Home Activities Recognition Rate (%)	87.06	81.18	77.59	68.72	73.82	74.84	92.33
Smart Office Activities Recognition Rate (%)	88.52	79.29	80.16	71.94	75.72	77.36	93.58
Smart Office Activities Recognition Rate (%)	84.47	75.46	72.02	63.28	67.15	72.04	90.33

**Table 13.** Confusion matrix of the proposed method using the MSRDailyActivity3D dataset. To have a clear view, we use two characters to represent each activity. Drink (DK), eat (ET), read book (RB), call on cellphone (CC), write on a paper (WP), use laptop (UL), use vacuum cleaner (UV), cheer up (CU), sit still (SS), toss paper (TP), play game (PG), lie down on sofa (LD), walk (WK), play guitar (PR), stand up (SU), and sit down (SD), respectively.

**Activities**	**DK**	**ET**	**RB**	**CC**	**WP**	**UL**	**UV**	**CU**	**SS**	**TP**	**PG**	**LD**	**WK**	**PR**	**SU**	**SD**
**DK**	**85.0**	5.0	0	0	0	0	0	0	0	0	5.0	0	5.0	0	0	0
**ET**	15.0	**70.0**	0	5.0	0	0	0	0	0	0	10.0	0	0	0	0	0
**RB**	0	5.0	**75.0**	0	10.0	0	5.0	0	0	0	0	0	0	5.0	0	0
**CC**	15.0	5.0	0	**65.0**	0	0	0	0	5.0	0	0	0	10.0	0	0	0
**WP**	0	0	20.0	0	**50.0**	15.0	0	0	10.0	0	5.0	0	0	0	0	0
**UL**	0	5.0	0	0	0	**80.0**	0	0	0	0	0	0	15.0	0	0	0
**UV**	0	0	5.0	0	0	0	**85.0**	0	0	0	0	5.0	0	5.0	0	0
**CU**	0	0	0	0	0	0	0	**90.0**	0	10.0	0	0	0	0	0	0
**SS**	0	0	0	0	5.0	0	0	0	**85.0**	0	10.0	0	0	0	0	0
**TP**	0	0	0	5.0	0	0	5.0	0	0	**75.0**	10.0	0	5.0	0	0	0
**PG**	0	0	5.0	0	0	0	0	0	15.0	0	**80.0**	0	0	0	0	0
**LD**	0	0	0	0	0	0	0	0	0	0	0	**90.0**	0	5.0	0	5.0
**WK**	0	0	0	0	0	0	5.0	0	0	0	0	0	**95.0**	0	0	0
**PR**	0	0	0	0	5.0	0	0	15.0	0	0	5.0	0	0	**75.0**	0	0
**SU**	0	0	0	0	0	0	0	0	10.0	0	0	0	0	0	**90.0**	0
**SD**	0	10.0	0	0	0	0	0	0	5.0	0	0	5.0	0	0	5.0	**75.0**

**Table 14.** Comparison of recognition accuracy on MSRDailyActivity3D dataset.

**Methods**	**Accuracy**
LOP Features [61]	42.5%
Dynamic Temporal Warping [62]	54.0%
Only Joint Position Features [61]	68.0%
SVM on Fourier Temporal Pyramid Features [61]	78.0%
Actionlet Ensemble Method [61]	85.7%
Multi-Fused Features Method [63]	93.1%
Proposed Motion Features	79.1%
